# A Tail-Anchored Myotonic Dystrophy Protein Kinase Isoform Induces Perinuclear Clustering of Mitochondria, Autophagy, and Apoptosis

**DOI:** 10.1371/journal.pone.0008024

**Published:** 2009-11-25

**Authors:** Ralph J. A. Oude Ophuis, Mietske Wijers, Miranda B. Bennink, Fons A. J. van de Loo, Jack A. M. Fransen, Bé Wieringa, Derick G. Wansink

**Affiliations:** 1 Department of Cell Biology, Nijmegen Centre for Molecular Life Sciences, Radboud University Nijmegen Medical Centre, Nijmegen, The Netherlands; 2 Rheumatology Research and Advanced Therapeutics, Nijmegen Centre for Molecular Life Sciences, Radboud University Nijmegen Medical Centre, Nijmegen, The Netherlands; Universidade Federal do Rio de Janeiro (UFRJ), Instituto de Biofísica da UFRJ, Brazil

## Abstract

**Background:**

Studies on the myotonic dystrophy protein kinase (DMPK) gene and gene products have thus far mainly concentrated on the fate of length mutation in the (CTG)n repeat at the DNA level and consequences of repeat expansion at the RNA level in DM1 patients and disease models. Surprisingly little is known about the function of DMPK protein products.

**Methodology/Principal Findings:**

We demonstrate here that transient expression of one major protein product of the human gene, the hDMPK A isoform with a long tail anchor, results in mitochondrial fragmentation and clustering in the perinuclear region. Clustering occurred in a variety of cell types and was enhanced by an intact tubulin cytoskeleton. In addition to morphomechanical changes, hDMPK A expression induces physiological changes like loss of mitochondrial membrane potential, increased autophagy activity, and leakage of cytochrome c from the mitochondrial intermembrane space accompanied by apoptosis. Truncation analysis using YFP-hDMPK A fusion constructs revealed that the protein's tail domain was necessary and sufficient to evoke mitochondrial clustering behavior.

**Conclusion/Significance:**

Our data suggest that the expression level of the DMPK A isoform needs to be tightly controlled in cells where the hDMPK gene is expressed. We speculate that aberrant splice isoform expression might be a codetermining factor in manifestation of specific DM1 features in patients.

## Introduction

The myotonic dystrophy protein kinase (*DMPK*) gene is involved in myotonic dystrophy type I (DM1) when it is mutant and contains an unstable (CTG)n segment in its 3′ terminal exon [1]. *DMPK* encodes several serine/threonine protein kinases, believed to be involved in ion homeostasis and remodeling of the actin cytoskeleton [2]–[5]. Up till now, emphasis in most DM1 studies was on the pathobiological significance of toxic RNA products from the mutant *DMPK* gene. Only relatively few studies have addressed individual protein products from the *DMPK* gene, including their normal structure function relationship [6], [7].

Constitutive and regulated modes of alternative splicing exist for DMPK pre-mRNA and result in the expression of six major DMPK splice isoforms, conserved between mouse and man. Individual isoforms are characterized by presence of either one of two types of long C-termini (tail versions 1 or 2; DMPK isoforms A to D) or a rather short C-terminus (tail 3; isoforms E and F), combined with absence or presence of an internal VSGGG-motif (A vs B, C vs D, E vs F) [5]. DMPK isoforms A–D are typical tail-anchored proteins with a membrane segment in their C-terminus. These isoforms are mainly expressed in heart, skeletal muscle and brain. Isoforms E and F are cytosolic proteins, predominantly found in smooth muscle cells [2], [5], [8].

Previously, we demonstrated that tail anchors in DMPK A/B and DMPK C/D drive binding to specific organellar membranes [9]. In mouse, this results in binding of mDMPK A and B to the endoplasmic reticulum (ER) and in binding of mDMPK C (and D) to the mitochondrial outer membrane (MOM). In humans, hDMPK A/B and C/D have also distinct tails, but these isoforms all anchor to the MOM. Isoform hDMPK A is unique in that its transient expression causes mitochondrial morphology to become abnormal, eventually leading to cell death via an as yet unidentified mechanism [10].

Mitochondria form an elaborate network with variable morphology and spatial distribution, tightly controlled by the physiological state of the cell and dependent on cell type and metabolic needs [11]. Organellar form and function in this network are regulated by fission and fusion with important bearing on the internal distribution of energy metabolites, the mode of sequestration of intracellular Ca^2+^ ions [12] and perhaps even apoptosis signaling [13]. MOM-associated proteins like mitofusins 1 and 2 (Mfn1 and 2) and OPA1 or hFis control mitochondrial fragmentation or perinuclear localization [14]–[17].

Various diseases, either coupled to acquired or inherited defects in bioenergetic circuits or to abnormalities in the fission-fusion machinery have been associated with abnormal mitophysiology [18]. Also in DM1 patients, abnormal mitochondrial form and mitochondrial dysfunction have been described [19], [20]. Furthermore, overexpression of RNA and protein products from a *hDMPK (CTG)11* transgene in a DM1 mouse model induced accumulation of mitochondria in the subsarcolemmal space and formation of aberrant cristae and caused a reduced workload tolerance in mice [21].

Quantitative and qualitative aspects of DMPK biology—via mitochondrial involvement—could thus contribute to typical features of DM1 disease manifestation, including defective Ca^2+^ ion homeostasis, insulin resistance and loss of cell viability in muscle, brain and other organs [7]. Here we report on one aspect, consequences of expression of the hDMPK A isoform, in particular its binding to the MOM. By use of transfection-complementation experiments in cultured cells, with or without DMPK deficiency, we analyze hDMPK A's role in determining mitochondrial fate and function and the functional integrity of the cell. We demonstrate that the protein's C-terminal tail is sufficient for induction of perinuclear clustering. Microscopy and biochemical analysis revealed that mitochondrial clustering was associated with increased autophagic activity. Ultimately, decoration of mitochondria with hDMPK A results in loss of cell-functional integrity and in the initiation of apoptosis.

## Results

### The tail region of hDMPK A is responsible for perinuclear mitochondrial clustering

**Figure 1:**
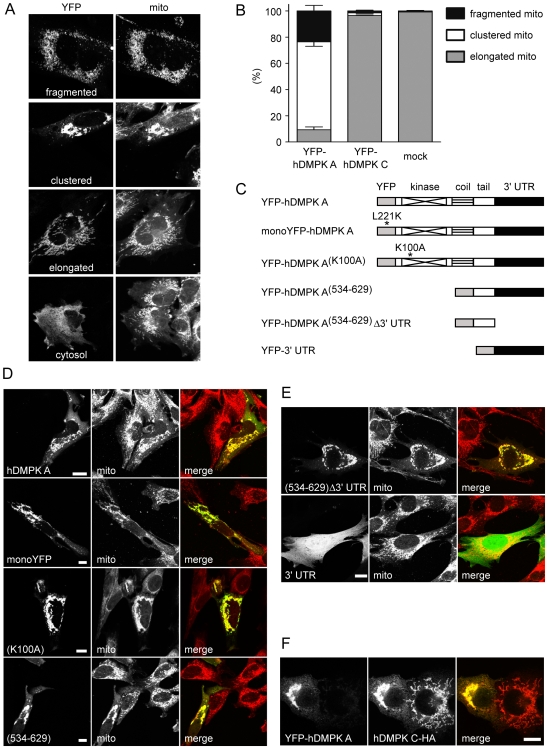
Perinuclear clustering of mitochondria is induced by the C-terminal domain of hDMPK A. (A,B) C_2_C_12_ myoblasts transiently expressing YFP-hDMPK A or C fusion proteins or mock-transfected cells were stained with a cytochrome c oxidase antibody to visualize mitochondria. Typical examples of classes of mitochondrial morphology are shown in (A). Mitochondrial distribution was classified as fragmented, clustered or elongated. Frequencies of appearance are listed as percentage of the total number of cells expressing MOM-associated hDMPK A (n = 3, ∼50 cells analyzed per experiment). Around 40% of YFP-hDMPK A-expressing cells showed a cytosolic expression. These cells contained mitochondria with a typically elongated shape, but were disregarded in the analysis. (C) Schematic representation of YFP constructs used for transfection expression with protein domains, mutations and 3′ UTR indicated. (D) C_2_C_12_ myoblasts expressing YFP fusion proteins were stained with a cytochrome c oxidase antibody to visualize their ability to induce clustering of mitochondria. (E) Expression of constructs with altered 3′ UTRs demonstrated that the hDMPK A 3′ UTR was not involved in mitochondrial clustering. (F) Co-expression of YFP-hDMPK A and hDMPK C-HA in N2A cells resulted in mitochondrial clustering, whereas cells only expressing hDMPK C-HA exhibited normal, elongated mitochondria. Bars, 10 µm.

Earlier, we had observed that hDMPK A has the capacity to localize to the MOM and affect mitochondrial distribution in a broad range of cell lines [10]. To analyze quantitative and qualitative aspects of this phenomenon in more detail, we used transfection of C_2_C_12_ myoblasts, a natural host cell line for DMPK proteins [8]. Transfection of YFP-hDMPK A resulted in mitochondrial decoration in ∼60% of all YFP-positive cells, while for the remaining ∼40% a cytosolic fluorescence without mitochondrial binding was observed. In contrast, mitochondrial YFP-fluorescence was found in virtually all cells that expressed YFP-hDMPK C. Classification of cells with MOM-bound hDMPK A (see Figure 1A for morphological classes) revealed that the majority of cells contained mitochondria with a clustered appearance (Figure 1B). Approximately 25% possessed fragmented mitochondria and only few showed a network of normal, elongated mitochondria. Frequencies in the different categories varied somewhat with experimental conditions, including transfection dose and duration (data not shown). Virtually all mitochondria in cells with cytosolic YFP-hDMPK A displayed a normal elongated appearance (Figure 1A, lowest panel), similar to cells with MOM-bound YFP-hDMPK C or mock-transfected cells. Taken together, these observations suggest that mitomorphological changes are only induced in cells where hDMPK A proteins are associated to mitochondria.

To investigate whether the entire protein or only domains of hDMPK A are involved in this phenomenon, a series of YFP-fusion constructs was designed (Figure 1C) and expressed in C_2_C_12_ myoblasts (Figure 1D). Tagging with monomeric L221K YFP variant was incorporated to exclude involvement of the dimerizing capacity of the YFP portion [9]. Use of this monomeric YFP tag did not affect hDMPK A's mitochondrial clustering behavior, demonstrating that this is indeed an intrinsic property of the DMPK moiety itself (Figure 1D). Importantly, mitochondrial clustering was still observed when the kinase-dead K100A mutant was expressed (Figure 1D), consistent with the idea that enzymatic activity is not required. Since hDMPK C also anchors at mitochondria but does not change mitochondrial morphology, even though it differs from hDMPK A only in the C terminus [10], we hypothesized that the tail domain must be responsible for mitochondrial clustering. Indeed, expression of mutant YFP-hDMPK A^(534–629)^ confirmed that clustering capacity was entirely contained within hDMPK A's C-terminus (Figure 1D).

It is well known that the 3′ UTR of a mRNA can determine gene product distribution and function in cells [22]. The 3′ UTR of hDMPK mRNA, when overexpressed, has been shown to be detrimental to cardiomyocytes and myofibers [23]. We therefore verified whether mitochondrial behavior differed between situations where hDMPK A protein was expressed from constructs with or without its normal 3′ UTR (Figure 1C and 1E), but found no differences. Also, a control vector containing a YFP ORF followed by the DMPK 3′ UTR gave the anticipated cytosolic and nuclear YFP distribution. Thus, our data indicate that the 3′ UTR is not involved in hDMPK A-induced mitochondrial clustering.

In most cell types where the *DMPK* gene is naturally expressed, isoforms A and C are present in approximately equal amounts [5,8, Mulders and Wansink, data not shown]. To test whether presence of hDMPK C could prevent hDMPK A from inducing mitochondrial clustering, we used N2A cells, well known for their high transfection efficiency, to generate sufficient numbers of cells that coexpressed hDMPK A and C in a double transfection. Isoform hDMPK C failed to modulate formation of mitochondrial clusters in all doubly transfected cells, indicating that hDMPK A effects are dominant (Figure 1F).

### Microtubule depolymerization reduces mitochondrial clustering

The cytoskeleton controls interactions in the mitochondrial network, facilitated by a host of membrane fission/fusion proteins, motor and adaptor proteins. Mitochondria are transported along microtubules and actin microfilaments [24]. Since hDMPK A appears to affect mitochondrial dynamics and is also believed to affect actomyosin behavior [6], [7], we reasoned that its role could be at the cytoskeletal-mitochondrial interface.

**Figure 2:**
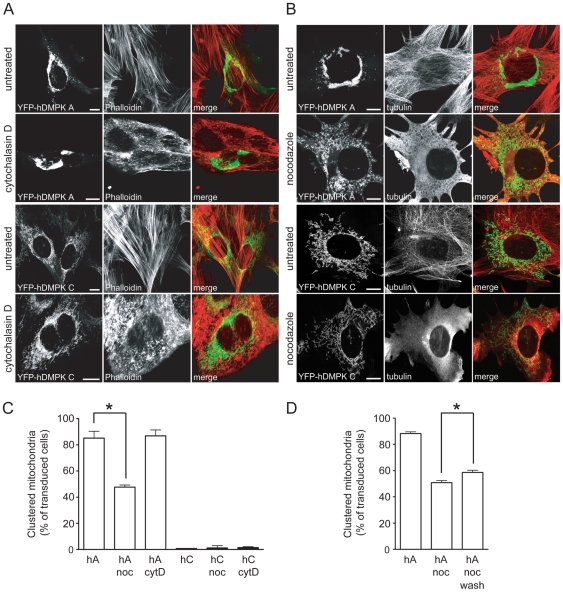
An intact microtubular cytoskeleton enhances hDMPK A–induced perinuclear mitochondrial clustering. *DMPK KO* myoblasts were transduced with YFP-hDMPK A or C-expressing adenoviruses in the presence of cytochalasin D (A) or nocodazole (B). F-actin was visualized by fluorescent phalloidin. The microtubular cytoskeleton was stained with an anti-tubulin antibody. Disruption of the actin cytoskeleton did not affect localization of mitochondria. Depolymerization of microtubules decreased mitochondrial clustering in YFP-hDMPK A-expressing cells, but mitochondria still appeared fragmented. The distribution of mitochondria in YFP-hDMPK C-transduced cells was unaffected by nocodazole treatment. Bars, 10 µm. (C) Quantification of mitochondrial clustering. The number of transduced cells that contain clustered mitochondria are expressed as percentage of the total amount of cells expressing hDMPK A at the MOM, with or without treatment of cytochalasin D or nocodazole (images shown in A and B; n = 3, ∼100 cells per experiment, P = 0.01). (D) Effect of nocodazole wash-out. Quantification of the percentage transduced cells with clustered mitochondria after a 12–16 hours treatment with nocodazole, followed by a 8 hours wash-out (n = 3, ∼30 cells per experiment, P<0.05).

Comparison of the actin and microtubule cytoskeletons in cells with YFP-hDMPK A or C expression did not reveal any overt effect on structural integrity (Figure 2A and 2B). We also examined what the absence of tubulin or actin filaments would do to the perinuclear accumulation of mitochondria [25], [26]. *DMPK* KO mouse myoblasts [10] were reconstituted with hDMPK A or C isoforms by adenoviral transduction and immediately treated with cytochalasin D or nocodazole. F-actin depolymerization-disorganization by cytochalasin D did not alter clustered mitochondrial morphology as revealed by comparison between YFP-hDMPK decoration patterns in A and C isoform-transfected cells (Figure 2A). Also the frequency of clustering was not altered by cytochalasin D treatment (measured as the percentage of transfected cells that displayed clustered mitochondria; Figure 2C). In contrast, in cells with a nocodazole-induced microtubular disorganization, the fraction of hDMPK A-expressing cells displaying perinuclear accumulation of mitochondria was significantly reduced, even though the fragmented appearance of the mitochondrial network remained (Figure 2B and 2C). When nocodazole was washed out at 12–16 hours after transduction and expression was continued for an additional 8 hours, the percentage of cells with mito-clusters was significantly higher than among cells that were continuously treated with nocodazole (Figure 2D). Nocodazole had no overt effect on mitochondrial location or morphology in hDMPK C-expressing cells. To us, these observations suggest that hDMPK A-induced fragmentation and clustering are at least partly uncoupled effects and that microtubular infrastructure only affects the extent of clustering.

### Mitochondrial morphology changes soon after hDMPK A translocation

**Figure 3:**
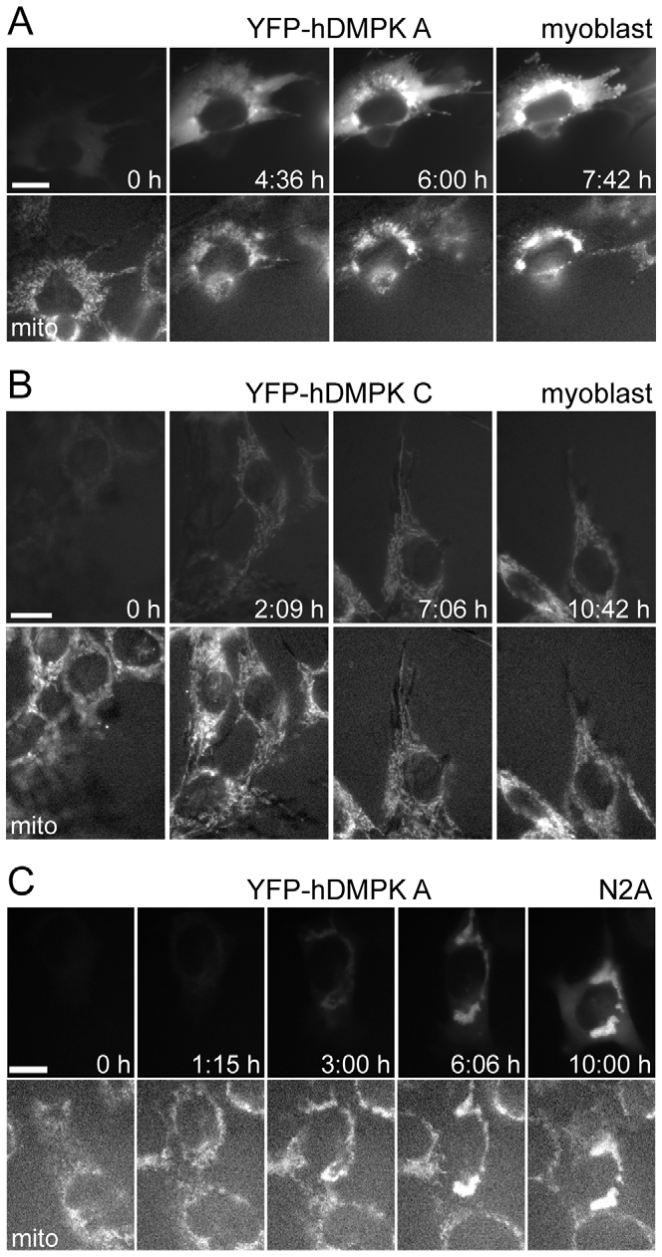
Mitochondrial clustering is a rapid process and occurs already at low hDMPK A expression. KO myoblasts were transduced with YFP-hDMPK fusion proteins (top panels), while mitochondria stained with MitoTracker Red (bottom panels). Images were collected every three minutes. The time of first appearance of YFP signal was set at t = 0; other time points are indicated in the top panels. (A) YFP-hDMPK A was first detected in the cytoplasm. Soon YFP-hDMPK–decorated mitochondria appeared which then started to cluster, eventually resulting in severely aggregated mitochondria surrounding the nucleus. (B) YFP-hDMPK C directly appeared on mitochondria which maintained their elongated, reticular structure. (C) In N2A cells, YFP-hDMPK A expression emerged in a similar fashion as in KO myoblasts, except that mitochondrial clustering occurred almost immediately and cytosolic staining was less pronounced.

To obtain insight in spatio-temporal aspects of mitochondrial morphology alterations, KO myoblasts were transduced with YFP-hDMPK isoforms and subjected to live imaging. Mitochondria were visualized via MitoTracker Red staining and images were taken every three minutes. YFP-hDMPK A located in the cytosol at first appearance, but translocated and concentrated at the MOM within four hours, after which fragmentation and clustering of mitochondria occurred (Figure 3A). YFP-hDMPK C appeared directly on mitochondria without inducing mitochondrial clusters (Figure 3B). In transfected N2A cells, initial YFP-hDMPK A staining in the cytosol was less evident and hDMPK A appeared at the MOM without intermediate accumulation, directly after expression onset. Size changes and clustering of mitochondria could be detected as early as six hours after transfection in these cells (Figure 3C). Combining these data with findings in other cell lines [10] demonstrates that timing of expression and sorting of DMPK isoforms may differ between cell types, but the sequence of events that is induced once DMPK A is associated with mitochondria is qualitatively similar.

### Abnormal mitochondrial ultrastructure develops upon hDMPK A expression

**Figure 4:**
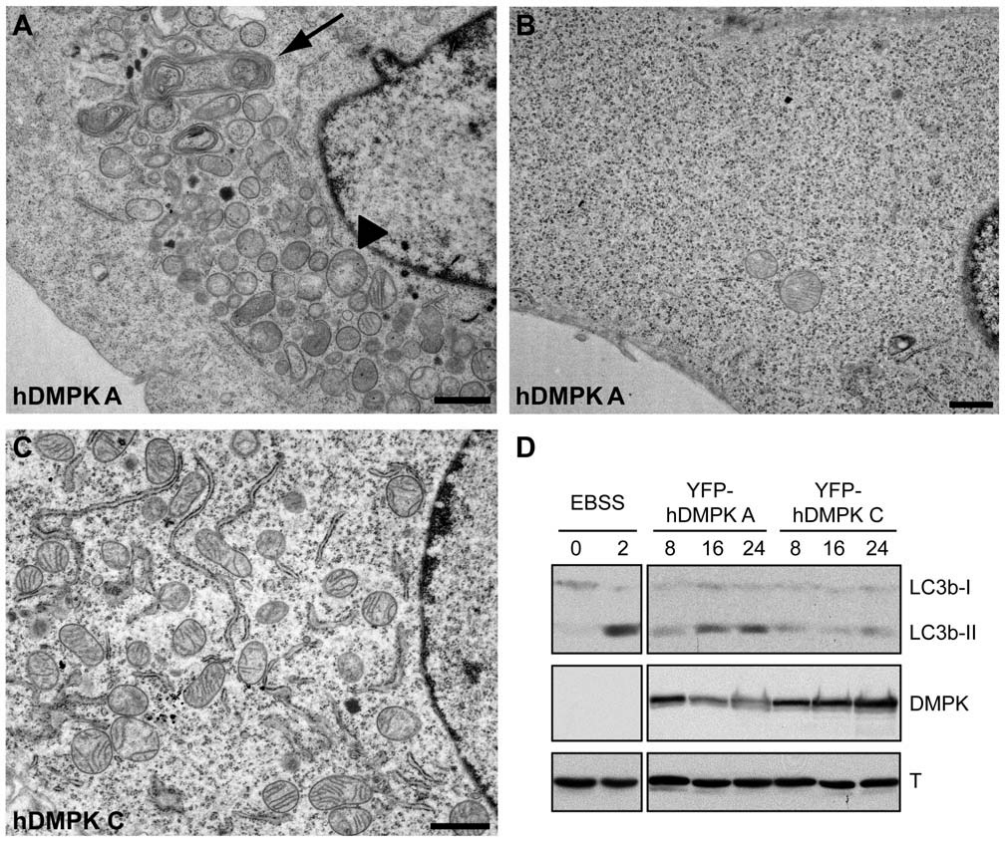
Mitochondrial clustering is accompanied by aberrant ultrastructural morphology. N2A cells were transfected with YFP-hDMPK A or C isoforms and ultrastructure was analyzed by electron microscopy. (A) YFP-hDMPK A–expressing cells contained fragmented, clustered mitochondria near the nucleus. Cristae structure was often partly lost together with electron density of the matrix (arrowhead) and mitophagy was observed (arrow). In contrast, other areas of the cell were completely devoid of mitochondria (B). (C) Mitochondrial morphology was normal in hDMPK C–expressing cells. A fragment of the nucleus is included here for orientation. Note the proper cristae structure and loose distribution of mitochondria and endoplasmic reticulum. Bars, 1 µm. (D) Lysates from myoblasts expressing YFP-hDMPK A or C for 8, 16 or 24 hours were used for western blotting with a LC3b antibody and showed an increased LC3b conversion following YFP-hDMPK A expression. Cells cultured under normal conditions and nutrient-starved cells, cultured in Earle's Balanced Salt Solution (EBSS) for 2 hours, were used as negative and positive controls, respectively. DMPK expression was verified using a DMPK antibody. Tubulin (T) antibody staining was used as loading control.

We performed electron microscopy (EM) on N2A cells expressing hDMPK A to investigate effects on mitochondrial ultrastructure and cell architecture. YFP-hDMPK A-transfected cells were easy recognizable by the concentration of mitochondria in the juxtanuclear region (Figure 4A), while other parts of the cell were left completely void of mitochondria (Figure 4B). Clustered mitochondria showed a rounded morphology, often with disorganized or absent cristae structure and a decrease of electron density in the matrix (Figure 4A). Cells with this appearance were never seen in YFP-hDMPK C-transfected cultures. In control cells or cells expressing YFP-hDMPK C mitochondria contained a normal, intact cristae structure, were more elongated and had a disperse localization (Figure 4C).

Besides abnormal mitochondria, we identified an increased number of autophagic structures after hDMPK A expression (Figure 4A). These autophagosomes varied in size and were found near and within areas of clustered mitochondria. In some cases, mitochondrial remnants could still be recognized, indicative of mitophagy. The fraction of cells containing autophagic structures was assessed by LM and EM and amounted to 12–14% after YFP-hDMPK A expression and 5–6% in cells with YFP-hDMPK C.

As a second indicator of autophagy, we measured conversion of LC3b protein using western blotting [27]. In transduced myoblasts significant conversion to the LC3b-II form was observed after 16–24 hours of expression of YFP-hDMPK A, but not C (Figure 4D). These observations were confirmed in N2A cells (data not shown). On blot, we repeatedly observed a reduced hDMPK A level within the 16–24 h period of expression. We attribute this finding to a selective loss of cells with highest DMPK A expression (see below). Although this may have influenced our picture of kinetics of autophagy induction in the entire pool of cells, our data collectively point to a gradual viability loss in the majority of cells in the transfected pool.

### Clustering of mitochondria results in loss of function

**Figure 5:**
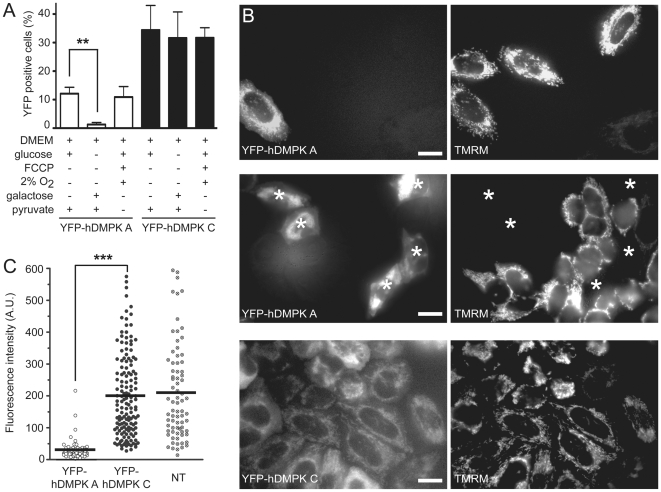
Human DMPK A expression affects mitochondrial function and cell viability. (A) C_2_C_12_ myoblasts were grown under different culture conditions and transfected with YFP-hDMPK A or C. The fraction of viable YFP positive cells was determined after 20 hours. When supplied with galactose and pyruvate, YFP-hDMPK A-expressing cells showed a significantly lower viability than YFP-hDMPK C-expressing cells (P<0.01, n = 3, >100 cells analyzed per experiment). (B) The MMP was determined in HeLa cells expressing YFP-hDMPK A or C. YFP-hDMPK A–expressing cells without clustered mitochondria showed a clear MMP signal (upper panels). No TMRM signal was found in YFP-hDMPK A–expressing cells with clustered mitochondria (middle panels, asterisks). YFP-hDMPK C–expressing cells demonstrated a clear mitochondrial signal (lower panels). Bars, 10 µm. (C) The MMP in YFP-hDMPK A–expressing cells was almost completely abolished and significantly lower than in YFP-hDMPK C and non-transfected (NT) cells (P<0.001, n = 3, >35 cells per experiment).

Against this background, knowing that mitochondrial integrity and function are dynamically linked and tightly coupled to the cell's physiological state [11], [28], we investigated hDMPK A effects on cell viability further. To force utilization of relevant metabolic energy pathways, C_2_C_12_ myoblasts were kept in media of different composition, including high or low oxygen supply, and monitored after transfection with YFP-hDMPK A or C. Cell survival was similar for YFP-hDMPK A-expressing cells growing under normal conditions or high glucose conditions with low oxygen plus use of mitochondrial uncoupler FCCP (to maximally prevent mitochondrial OXPHOS activity; Figure 5A). This outcome can be explained because normal growth of cells in culture already strongly relies on glycolytic metabolism [29] and therefore cannot be further promoted. In glucose-deprived medium supplemented with galactose, forcing cells to rely on mitochondrial oxidative phosphorylation [30], hardly any YFP-hDMPK A-expressing cell survived (Figure 5A). This suggests that clustering of mitochondria is detrimental for cell viability, especially in aerobically-poised situations when cells are forced to use mitochondrial TCA/OXPHOS activity. As a control, YFP-hDMPK C-expressing cells showed no differences in cell survival under these culture conditions (Figure 5A).

Formation of an electrochemical proton gradient across the inner mitochondrial membrane drives ATP production by OXPHOS [31]. To compare possible effects of DMPK isoforms on this coupling, we used staining of YFP-hDMPK A and C-expressing HeLa cells with the fluorescent dye TMRM, which provides a readout for the mitochondrial membrane potential (MMP). HeLa cells were chosen because these have been used before for MMP determination and gave sufficient TMRM signal strength for quantitative analysis [32]. Before measurement, depolarization with FCCP as described by Distelmaier et al. [33] was used to control that TMRM accumulation did not involve quenching, becoming a confounding factor in MMP measurement. For many other cell lines the 100 nM TMRM concentration appeared toxic or TMRM did not accumulate well enough in mitochondria to generate adequate signals (data not shown). YFP-hDMPK A-expressing HeLa cells showed a significantly lower MMP than cells that expressed YFP-hDMPK C (Figure 5B and 5C). Interestingly, YFP-hDMPK A-positive cells with no or only mild mitochondrial clustering showed a normal MMP (Figure 5B, upper panels). The lack of signal in clustered mitochondria could not be due to an inability to take up TMRM, since they were able to internalize the related compound MitoTracker Red (data not shown). The MMP in YFP-hDMPK C-expressing cells was not significantly different from that in untransfected cells (Figure 5C).

### Cells with clustered mitochondria undergo apoptosis

**Figure 6:**
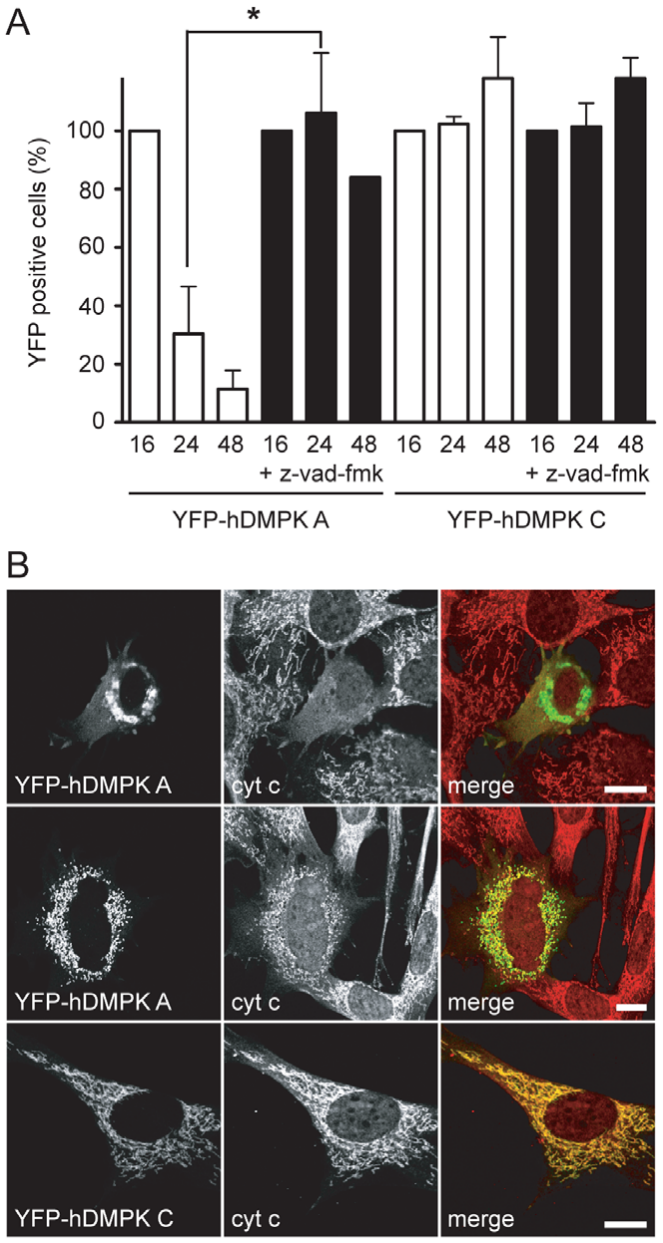
Expression of hDMPK A induces apoptosis. (A) The effect of apoptosis-inhibitor z-vad-fmk was tested on cell survival of DMPK KO myoblasts transduced with YFP-hDMPK A or C–expressing adenoviruses. YFP-positive cells were counted after 16, 24, and 48 hours. Z-vad-fmk greatly reduced cell death of YFP-hDMPK A–expressing cells, but had no effect when YFP-hDMPK C was expressed. Z-vad-fmk was applied immediately after transduction and maintained present for the remaining time of the experiment. Values at 16 hours were set at 100% (P<0.05, n = 3, >90 cells counted per experiment). (B) YFP-hDMPK A and C–expressing cells were stained for cytochrome c (cyt c). Cells expressing YFP-hDMPK A displayed a diffuse, cytosolic cytochrome c staining when mitochondria were clustered (upper panels). A clear mitochondrial staining was present when mitochondria appeared fragmented (middle panels). A discrete, mitochondrial cytochrome c staining was also observed in YFP-hDMPK C–expressing cells. Bars, 10 µm.

We observed that YFP-hDMPK A-expressing KO myoblasts - but not YFP-hDMPK C expressing cells - died rapidly after prolonged periods of transfection-complementation and wondered whether this loss could be explained by an apoptosis-based mechanism. One finding supporting this conjecture was that treatment with apoptosis inhibitor z-vad-fmk immediately after transfection significantly increased the number of surviving hDMPK A-expressing cells, whereas no effect was seen on the viability of cells expressing hDMPK C (Figure 6A). Further support was obtained by the observation that little or no staining of cytochrome c, a mitochondrial intermembrane protein, was present in clustered mitochondria in YFP-hDMPK A-expressing KO myoblasts (Figure 6B, upper panels), while mitochondria that were only fragmented did contain cytochrome c (Figure 6B, middle panels). To us this suggests that release of cytochrome c, a well known apoptosis-triggering event [34], probably occurs after the onset of mitochondrial clustering. No cytochrome c release was observed for YFP-hDMPK C-expressing cells (Figure 6B, lower panels).

## Discussion

We demonstrate here that expression of MOM-anchored hDMPK A results in fragmentation of the mitochondrial network and perinuclear clustering of morphologically-altered mitochondria. In addition to these morphomechanical changes, physiological changes like loss of MMP, increased autophagosomal activity and leakage of cytochrome c accompanied by apoptosis were induced. It is difficult to bring hierarchical ordering in these events, but we propose that the fate of mitochondria in cells with hDMPK A is specified as follows: based on our observations with live-cell imaging and the finding that microtubule disruption attenuated clustering, we suppose that induction of morphological change is the initiating step that marks mitochondria for subsequent anomalous transport and clustering. Upregulation of autophagy may be an intermediate phase to rescue defective energy and clear mitochondrial waste [35].

The fact that the same sequence of events in mitochondrial fragmentation and perinuclear clustering with globally similar final outcome occurred in all mouse and human cell types examined is important. Slight variation in the speed and magnitude of events was observed between cell types, but this can most easily be explained by differences in cytoarchitectural arrangement, pathophysiological thresholds or stress-coping ability. Toxicity of hDMPK A was also observed in KO myoblasts in which DMPK was reconstituted to apparently normal expression levels by complementation. We observed mitochondrial clustering already after several hours of hDMPK A expression when ectopic protein levels were still relatively moderate. Therefore, we postulate that induction of abnormal mitochondrial behavior is an intrinsic capacity of hDMPK A and cannot be simply explained as a result of overexpression. Mutational analysis demonstrated that hDMPK A's tail region was sufficient to induce mitochondrial fragmentation and clustering. The conclusion that this is the only segment with detrimental effects was corroborated by the observation that MOM-binding of hDMPK C, which differs from hDMPK A only in its C-terminus, had no effect on mitochondrial physiology and trafficking.

Interesting parallels can be drawn to the morphophysiological effects induced by ectopic or overexpression of other MOM proteins, like TA proteins hFis [15], [36], Miro GTPases [37], [38] and Mfn 1 and 2 proteins [14], [17], [39]. All these enzymes are involved in mitochondrial dynamics. Although similar in nature, changes evoked by these MOM proteins cannot be simply lumped together, since it is unclear how mitochondrial clustering occurs, and clear mechanistic differences can be noted. For example, enzymatic activity appears irrelevant for Mfn 1 and 2 [14], [17], [39] and hDMPK A (this paper), but is necessary for hFis [36] and Miro 1 and 2 [37]. We demonstrate that hDMPK A effects do not require coiled-coil interactions between proteins, but Mfn 1 and 2 effects clearly rely on these associations [40]. The status of the cytoskeleton does not determine Mfn 2 effects [14], yet hDMPK A clustering partly depends on integrity of microtubules. It is tempting to speculate that every distinct TA-containing MOM protein has its own role in creating an appropriate microenvironment for mitochondrial interactions with other cell structures. Reactions mediated by these proteins may involve distinct areas in the MOM and also the complex and diverse machinery that links mitochondria to motor proteins may be differentially involved [37], [38], [41], [42]. Disbalance in any of these systems may create overtly similar effects, with fragmentation and clustering of mitochondria as main outcome.

Mitochondrial fragmentation and clustering is probably an intrinsic property of the hDMPK A membrane anchor itself. There is mounting evidence indicating that certain hydrophobic regions in membrane proteins are able to induce membrane curvature and remodeling. Our EM data demonstrate that hDMPK A-decorated mitochondria are small and round - i.e., not one large continuum - suggesting that lipid-protein or lipid-lipid interactions become abnormally affected in presence of the tail anchor of hDMPK A. Direct effects of membrane anchors that result in distortion of protein-protein interaction and clustering followed by formation of abnormal membrane curvature, vesicle formation or fragmentation have been described [43], [44]. Coupling to abnormal fusogenic properties of hDMPK A's anchor could also be involved. It has been shown that Leu/Val residues and Gly/Pro or Pro/Gly pairs in peptides in transmembrane domains add to a high fusogenic index [45], [46]. Exactly these amino acids are highly abundant in the tail region of hDMPK A, but not hDMPK C. Further knowledge about involvement of lipid interactions with specific peptide sequences in hDMPK A's tail can be obtained by permutation analyses and biophysical interaction studies. At this moment we can only remain speculative about the importance of the amino acid composition of the membrane anchor.

Many studies have shown that the physiological state of mitochondria is reciprocally coupled to morphological appearance [17], [47]. Recently, even a relationship between mitochondrial form and cell cycle control has been reported [48]. Although mitochondrial fragmentation has been observed frequently in cells undergoing apoptosis, its active role in apoptosis is still unclear [36], [49]. We observed that hDMPK A-induced morphological effects ultimately lead to cell death and provide evidence that cytochrome c leak did not occur and MMP was still largely intact in hDMPK A-decorated mitochondria that were still in the fragmented state. Loss of cytochrome c and disappearance of MMP was observed when mitochondria became clustered. We take these observations to indicate that events that occur during or just before mitochondrial clustering must contribute to the onset of apoptosis.

Aberrant mitochondrial morphology is linked to multiple disease manifestations caused by mutations in genes involved in fission and fusion, like Charcot-Marie-Tooth subtype 2A and dominant optic atrophy [18], [28]. Also in DM1 patient muscle, mitochondrial irregularities have been found, although the cause of these malformations has not yet been identified [19], [20]. Studies on Tg26-*hDMPK* mice, overexpressing tail-anchored hDMPK isoforms especially in heart and skeletal muscle, showed disorganized cristae structure and symptoms like reduced workload tolerance, atrophy, hypertrophic cardiomyopathy, myotonic myopathy and hypotension, reminiscent of DM1 symptoms [21].

We do not expect that the normal physiological function of hDMPK is directly related to regulation of mitochondrial clustering. Like hDMPK C, hDMPK A is predominantly expressed in skeletal muscle myofibers [5], [8], a cell type with a rigidly compartmentalized cytoarchitectural infrastructure and well-defined mitochondrial distribution across intramyofibrillar and subsarcolemmal areas. This typical robust cytoarchitectural organization may render muscle cells more resistant to fluctuations in hDMPK A expression, but based on our findings we speculate that also for myotubes it is of paramount importance that hDMPK A protein levels are tightly regulated and strictly kept within the normal physiological range. Expression levels of completely other proteins, for example VEGF, also require tight regulation as both reduced and increased expression can result in pathogenesis, manifesting itself in embryonic lethality or cancer, respectively [50]. Changes in DMPK mRNA synthesis or pre-mRNA splicing induced by the presence of expanded (CUG)n transcripts, when resulting in only a slight disbalance in hDMPK A expression level, could thus be a contributing factor in the pathophysiology of DM and explain mitochondrial abnormalities. In more extreme situations like congenital DM with very large (CTG)n expansions, where abnormal chromatin modification and defective bidirectional transcriptional control across the DM locus may directly couple to unbalanced DMPK production, the toxic effects of DMPK A may even directly contribute to special disease features [51], [52]. Further research into the role of hDMPK A in health and disease is thus warranted and also needed to validate this model and its predictions. A more precise study of the balance in DMPK splice isoforms in different categories of DM1 patients is a necessary first step.

## Materials and Methods

### Antibodies and chemicals

Staining with anti-cytochrome c oxidase antibody [53] was used to visualize mitochondria and monitor apoptosis. Monoclonal β-tubulin antibody E7 (Developmental Studies Hybridoma Bank, University of Iowa, Iowa City, Iowa) was used to stain the tubulin cytoskeleton. TexasRed-conjugated-phalloidin staining (Molecular Probes, Breda, The Netherlands) was used to visualize F-actin. Manipulation of the cytoskeleton was performed by incubating cells for 18 hours in cell culture medium supplemented with 3 µM nocodazole (Sigma, Zwijndrecht, The Netherlands) or 0.6 µM cytochalasin D (Sigma). Mouse monoclonal antibody 12CA5 was used to detect the HA epitope tag. For western blotting DMPK-specific antibody B79 [5], β-tubulin antibody E7 and LC3b polyclonal antibody (Cell Signalling, Beverly, Massachusetts) were used. Apoptosis was inhibited by addition of 100 µM of the pan-caspase inhibitor z-vad-fmk (R&D systems, Abingdon, UK) to the culture medium for 16–72 hours.

### Cell culture and DNA transfection

C_2_C_12_ myoblasts (ATCC #CRL-1772), N2A neuroblastoma cells (ATCC #CCL-131) and HeLa cells (ATCC #CCL-2) were grown subconfluent in DMEM supplemented with 10% FCS and maintained at 37°C under a 5% CO_2_ atmosphere. C_2_C_12_ and N2A cells were transiently transfected with expression plasmids (specified below) using Lipofectamine (Invitrogen, Breda, the Netherlands) as specified by the manufacturer. HeLa cells were transiently transfected using polyethyleneimine. After transfection cells were maintained in culture for an additional 8–72 h prior to analysis. Alternatively, we used adenoviral vector-based DNA transduction for expression of single hDMPK isoforms in *DMPK* knockout (KO) myoblasts [10], since transfection with lipofectamine or polyethyleneimine does not achieve sufficient efficiency.

To force C_2_C_12_ myoblasts to use mitochondrial oxidative phosphorylation for the production of ATP, cells were cultured in DMEM without glucose, supplemented with 10 mM galactose, 1 mM sodium pyruvate, 2 mM glutamine and 10% dialyzed FCS. Cells were maintained at 37°C under a 5% CO_2_ atmosphere. To maximally stimulate glycolytic metabolism, C_2_C_12_ myoblasts were cultured in DMEM, 10% FCS with 10 mM glucose and 1 µM of the mitochondrial uncoupler FCCP (Sigma, Zwijndrecht, The Netherlands) and maintained at 37°C under a 2% O_2_ atmosphere. Cells were allowed to adapt to these culture conditions for 5 days, transfected with equal amounts of plasmid DNA encoding YFP-hDMPK A or C and then maintained in culture for an additional 20 hours before the number of YFP-positive cells was analyzed.

Autophagy was induced by placing cells in nutrient-starvation medium Earle's Balanced Salt Solution (EBSS) for two hours.

### Plasmids and adenoviral vectors for DMPK expression

EYFP-DMPK expression vectors were obtained by cloning appropriate DMPK cDNA segments into pEYFP-C1 (Clontech, Saint-Germain-en-Laye France) and pSG8ΔEco vectors [2], [10]. Inserts of all expression plasmids were composed of relevant domains from the open reading frames plus the adjacent 3′ UTR of DMPK cDNAs. All segments obtained via PCR were verified by DNA sequencing.

#### MonoYFP-hDMPK A

To introduce a L221K mutation into YFP, pEYFP-C1 vector was PCR-amplified with forward primer 5′-GATCACATGGTCCTTAAGGAGTTCGTGACC-3′ and reverse primer 5′- GGTCACGAACTCCTTAAGGACCATGTGATC-3′ (mutation underlined). The resulting fragment was digested with *BsrG*I and *Nhe*I, gel purified and subcloned into pEYFP-C1. A *Bgl*II excised hDMPK A fragment was introduced into the *Bgl*II site.

#### YFP-hDMPK A^(K100A)^

A PCR fragment was amplified by site-directed mutagenesis from a template pEYFP-hDMPK A plasmid with forward primer 5′- GGCCAGGTGTATGCCATGGCAATCATGAACAAGTGGGAC-3′ and reverse primer 5′- GTCCCACTTGTTCATGATTGCCATGGCATACACCTGGCC -3′ (mutation underlined). The resulting PCR product was digested with *BspE*I and the fragment containing the mutation was subcloned between two *BspE*I sites of pEYFP-hDMPK A.

#### YFP-hDMPK A^(534–629)^

A fragment encompassing human tail 1 including the 3′ UTR was PCR-amplified from pEYFP-hDMPK A with forward primer 5′-GGAAGATCTGCTGTCACGGGGGTCCC-3′ adding a *Bgl*II site (underlined) and reverse primer 5′- TGCAATAAACAAGTTAACAACAAC-3′ located in the multiple cloning site of the pEYFP-C1 backbone. The resulting DNA was trimmed with *Bgl*II, gel purified and subcloned into the polylinker of vector pEYFP-C1.

#### YFP-hDMPK A^(534–629)^Δ3′ UTR

A fragment encompassing human tail 1 excluding the 3′ UTR was PCR-amplified from pEYFP-hDMPK A with forward primer 5′-GGAAGATCTGCTGTCACGGGGGTCCC-3′ adding a *Bgl*II site (underlined) and reverse primer 5′-CGAATTCTCAGGGAGCGCGGGCGG-3′ located at the stop codon containing an *Eco*RI site (underlined). The resulting DNA was trimmed with *Bgl*II and *Eco*RI, gel purified and subcloned between the *Bgl*II and *Eco*RI restriction sites in the polylinker of pEYFP-C1.

#### YFP-3′ UTR

A fragment encompassing the hDMPK A 3′ UTR was PCR-amplified from pEYFP-hDMPK A, with forward primer 5′-GGAAGATCTTGAACCCTAGAACTGTCTTC-3′ adding a *Bgl*II site (underlined) and reverse primer 5′-TGCAATAAACAAGTTAACAACAAC-3′ located in the multiple cloning site of the pEYFP-C1 backbone. The resulting DNA was trimmed with *Bgl*II, gel purified and subcloned into the polylinker in vector pEYFP-C1.

#### hDMPK C-HA

A fragment encompassing the final 900 bp of the coding region of hDMPK C was amplified by PCR from pEYFP-hDMPK C with forward primer 5′-GCCGCTGGTGGACGAAGGG-3′ and reverse primer 5′-GCCGGTAC*C* *TAAGCGTAATCTGGAACATCGTATGGGTA*GGGTTCAGGGAGCGCGG-3′ adding an HA tag (italics) and *Kpn*I site (underlined). The PCR fragment was trimmed with *Sst*I and *Kpn*I resulting in a 500 bp fragment. A fragment of about 1.1 kb encompassing the 3′ UTR of hDMPK C was PCR amplified with forward primer 5′-GCCGGTACCTGAACCCTAGAACTGTCTTCG-3′ adding a *Kpn*I site (underlined) and reverse primer 5′-TGCAATAAACAAGTTAACAACAAC-3′ located in the multiple cloning site of pEYFP-C1. The PCR product was trimmed with *Kpn*I and *Sal*I resulting in a 800 bp fragment. The two fragments were subcloned into pEYFP-hDMPK C digested with *Sst*I and *Sal*I. The pEYFP-hDMPK C-HA construct was digested with *Bgl*II and ligated into the *Bgl*II sites of pSG8ΔEco resulting in pSG8ΔEco-hDMPK C-HA.

E1/E3-deleted serotype 5 adenoviral vectors encoding YFP-hDMPK isoforms A and C under the control of a CMV IE-promoter were generated using the AdEasy Vector System [54], as described [55]. In brief, cDNAs encoding YFP-hDMPK isoforms including their 3′-UTR, were obtained from pEYFP-hDMPK A or C and cloned into transfer vector pShuttle-CMV. In a second step, pShuttle-YFP-hDMPK plasmid was recombined with the viral DNA plasmid pAdEasy-1 in *E. Coli* strain BJ5183. Next, viral particles were generated in N52.E6 [56] viral packaging cells [57]. Adenoviral vectors were purified using CsCl gradient purification and stored at −80°C. The viral titer and plaque forming units were determined [55].

### Immunofluorescence microscopy

Cells were grown on glass coverslips, fixed in PBS containing 2% (w/v) formaldehyde ∼20 h after transfection and permeabilized in PBS containing 0.5% (w/v) Nonidet P-40 substitute. Samples were processed for immunofluorescence microscopy using standard procedures. Images were obtained with a Bio-Rad MRC1024 confocal laser scanning microscope (Biorad, Hercules, California) equipped with an argon/krypton laser, using a 60x 1.4 NA oil objective and LaserSharp2000 acquisition software.

### Time-lapse imaging

Transfected KO myoblasts or N2A cells were stained for 30 minutes using 1 µM MitoTracker Red (CM-H_2_XROS; Invitrogen). After washing with acquisition medium (DMEM without phenol red supplemented with 10% (N2A) or 20% FCS (KO myoblasts)), dishes were mounted into a temperature-controlled incubation chamber on the stage of an inverted microscope (Axiovert 200 M; Carl Zeiss, Jena, Germany) equipped with a 63x, 1.25 NA Plan NeoFluar oil-immersion objective. Images were acquired every three minutes in three Z-directions at 33°C for myoblasts and 37°C for N2A cells during which cells were maintained in standard culture medium without phenol red. YFP was excited at 510 nm with an acquisition/illumination time of 200 ms and fluorescence light was directed through a 545AF35 emission filter (Omega Optical, Brattleboro, VT). MitoTrackerRed was excited at 568 nm (Polychrome IV) with an acquisition/illumination time of 200 ms and fluorescence light was directed by a 525DRLP dichroic mirror (Omega) through a 564AF65 emission filter (Omega). A CoolSNAP HQ monochrome CCD-camera (Roper Scientific Photometrics, Vianen, The Netherlands) was used and no bleed through was detected. Hardware was controlled by and images were analyzed using Metamorph 6.2 software for mean fluorescence intensity of the mitochondrial area.

### Western blotting

Cells were lysed on ice in NP40 lysis buffer (1% NP40, 50 mM Tris-HCl, pH 7.5, 150 mM NaCl, 25 mM NaF, 1 mM sodium pyrophosphate, 0.1 mM vanadate, 1 mM PMSF, 1x protease inhibitor cocktail (Roche, Mannheim, Germany), 1 mM EDTA). Lysates were cleared by centrifugation for 10 min at 14,000 g at 4°C and supernatant fractions were mixed with SDS-PAGE sample buffer. Lysates were separated on 8% SDS-PAGE gels and transferred by western blotting to PVDF membrane. As secondary antibody, HRP-conjugated IgG (Jackson ImmunoResearch Laboratories, UK) was used, and signals were generated by ECL, followed by exposure to film (Kodak X-OMAT AR).

### Mitochondrial membrane potential (MMP)

HeLa cells were cultured in Willco dishes (Willco wells B.V., Amsterdam, The Netherlands) and transfected with pEYFP-hDMPK A and pEYFP-hDMPK C. After ∼20 h, cells were stained for 30 minutes using tetramethylrhodamine methyl ester TMRM (Invitrogen) at 100 nM in DMEM supplemented with 10% FCS. Wilco dishes were mounted on the stage of the inverted microscope. Experiments were performed at room temperature during which cells were maintained in phenol red-free DMEM supplemented with 10% FCS. TMRM was excited at 555 nm with an acquisition/illumination time of 100 ms using a monochromator (Polychrome IV, TILL Photonics, Gräfelfing, Germany) and fluorescence light was directed by a 560DRLP dichroic mirror (Omega) through a 565ALP emission filter (Omega) and detected as above. YFP was excited at 470 nm (Polychrome IV) with an acquisition/illumination time of 100 ms and fluorescence light was directed by a 505DRLPXR dichroic mirror (Omega) through a 565ALP emission filter. No bleed-through was detected. All hardware was controlled using Metafluor 6.0 software (Molecular Devices Corporation, Downingtown, PA). Images were analyzed using Metamorph 6.2 software for mean fluorescence intensity of the mitochondrial area [58].

### Electron microscopy

Cells were fixed in 2% glutaraldehyde in 0.1 M cacodylate buffer and post-fixed for 1 h in 1% osmium tetroxide and 1% potassium ferrocyanide in 0.1 M cacodylate buffer. After washing in buffer, cells were dehydrated in an ascending series of aqueous ethanol and transferred via a mixture of ethanol and Epon to pure Epon 812 as embedding medium. Ultrathin sections (60–80 nm) were cut, contrasted with aqueous 3% uranyl acetate, rinsed, and counterstained with lead citrate, air dried, and examined in a JEOL JEM1010 electron microscope (JEOL, Welwyn Garden City, UK) operating at 80 kV.

### Autophagosome quantification by LM and EM

Samples were fixed and embedded as described for EM analysis. 1% osmium tetroxide was included to increase visibility of membranes [59]. Sections of 0.5 µm were cut and stained with toluidine blue. Sections were mounted on a cover glass using Eukitt (Sigma-Aldrich) and analyzed using light microscopy with a Leica DM6000B/CTR6000 imaging system and Leica IM500 Image Manager acquisition software (Leica, Solms, Germany). The percentage of cells containing one or more spheroid autophagosome structures was quantified. Quantification on ultrathin sections by electron microscopy was done in a similar manner.

### Statistics

Data are expressed as mean ± s.e.m. Between group comparison was performed by two-tailed unpaired Student's t-test. Differences between groups were considered significant when P<0.05. * P<0.05, ** P<0.01, *** P<0.001. Statistical analyses were performed with GraphPad Prism 4 software. The number of replicates and the number of cells counted per experiment are described in the figure legends.

## References

[1] Mahadevan MS, Amemiya C, Jansen G, Sabourin L, Baird S (1993). Structure and genomic sequence of the myotonic dystrophy (DM kinase) gene.. Hum Mol Genet.

[2] Wansink DG, van Herpen RE, Coerwinkel-Driessen MM, Groenen PJ, Hemmings BA (2003). Alternative splicing controls myotonic dystrophy protein kinase structure, enzymatic activity, and subcellular localization.. Mol Cell Biol.

[3] Benders AA, Groenen PJ, Oerlemans FT, Veerkamp JH, Wieringa B (1997). Myotonic dystrophy protein kinase is involved in the modulation of the Ca^2+^ homeostasis in skeletal muscle cells.. J Clin Invest.

[4] Mounsey JP, John JE,, Helmke SM, Bush EW, Gilbert J (2000). Phospho-lemman is a substrate for myotonic dystrophy protein kinase.. J Biol Chem.

[5] Groenen PJ, Wansink DG, Coerwinkel M, van den Broek W, Jansen G (2000). Constitutive and regulated modes of splicing produce six major myotonic dystrophy protein kinase (DMPK) isoforms with distinct properties.. Hum Mol Genet.

[6] Wansink DG, van Herpen REMA, Wieringa B, Wells RD, Ashizawa T (2006). Normal and pathophysiological significance of myotonic dystrophy protein kinase.. Genetic Instabilities and Neurological Diseases. 2nd ed.

[7] Kaliman P, Llagostera E (2008). Myotonic dystrophy protein kinase (DMPK) and its role in the pathogenesis of myotonic dystrophy 1.. Cell Signal.

[8] Oude Ophuis RJ, Mulders SA, van Herpen RE, van de Vorstenbosch R, Wieringa B (2009). DMPK protein isoforms are differentially expressed in myogenic and neural cell lineages.. Muscle Nerve.

[9] Snapp EL, Hegde RS, Francolini M, Lombardo F, Colombo S (2003). Formation of stacked ER cisternae by low affinity protein interactions.. J Cell Biol.

[10] van Herpen RE, Oude Ophuis RJ, Wijers M, Bennink MB, van de Loo FA (2005). Divergent mitochondrial and endoplasmic reticulum association of DMPK splice isoforms depends on unique sequence arrangements in tail anchors.. Mol Cell Biol.

[11] Benard G, Rossignol R (2008). Ultrastructure of the mitochondrion and its bearing on function and bioenergetics.. Antioxid Redox Signal.

[12] Spat A, Szanda G, Csordas G, Hajnoczky G (2008). High- and low-calcium-dependent mechanisms of mitochondrial calcium signalling.. Cell Calcium.

[13] Youle RJ, Karbowski M (2005). Mitochondrial fission in apoptosis.. Nat Rev Mol Cell Biol.

[14] Rojo M, Legros F, Chateau D, Lombes A (2002). Membrane topology and mitochondrial targeting of mitofusins, ubiquitous mammalian homologs of the transmembrane GTPase Fzo.. J Cell Sci.

[15] Yoon Y, Krueger EW, Oswald BJ, McNiven MA (2003). The mitochondrial protein hFis1 regulates mitochondrial fission in mammalian cells through an interaction with the dynamin-like protein DLP1.. Mol Cell Biol.

[16] Satoh M, Hamamoto T, Seo N, Kagawa Y, Endo H (2003). Differential sublocalization of the dynamin-related protein OPA1 isoforms in mitochondria.. Biochem Biophys Res Commun.

[17] Huang P, Yu T, Yoon Y (2007). Mitochondrial clustering induced by overexpression of the mitochondrial fusion protein Mfn2 causes mitochondrial dysfunction and cell death.. Eur J Cell Biol.

[18] Knott AB, Perkins G, Schwarzenbacher R, Bossy-Wetzel E (2008). Mitochondrial fragmentation in neurodegeneration.. Nat Rev Neurosci.

[19] Ueda H, Shimokawa M, Yamamoto M, Kameda N, Mizusawa H (1999). Decreased expression of myotonic dystrophy protein kinase and disorganization of sarcoplasmic reticulum in skeletal muscle of myotonic dystrophy.. J Neurol Sci.

[20] Siciliano G, Mancuso M, Tedeschi D, Manca ML, Renna MR (2001). Coenzyme Q10, exercise lactate and CTG trinucleotide expansion in myotonic dystrophy.. Brain Res Bull.

[21] O'Cochlain DF, Perez-Terzic C, Reyes S, Kane GC, Behfar A (2004). Transgenic overexpression of human DMPK accumulates into hypertrophic cardiomyopathy, myotonic myopathy and hypotension traits of myotonic dystrophy.. Hum Mol Genet.

[22] Czaplinski K, Singer RH (2006). Pathways for mRNA localization in the cytoplasm.. Trends Biochem Sci.

[23] Mahadevan MS, Yadava RS, Yu Q, Balijepalli S, Frenzel-McCardell CD (2006). Reversible model of RNA toxicity and cardiac conduction defects in myotonic dystrophy.. Nat Genet.

[24] Chang DT, Reynolds IJ (2006). Mitochondrial trafficking and morphology in healthy and injured neurons.. Prog Neurobiol.

[25] Anesti V, Scorrano L (2006). The relationship between mitochondrial shape and function and the cytoskeleton.. Biochim Biophys Acta.

[26] Yaffe MP, Harata D, Verde F, Eddison M, Toda T (1996). Microtubules mediate mitochondrial distribution in fission yeast.. Proc Natl Acad Sci U S A.

[27] Karim MR, Kanazawa T, Daigaku Y, Fujimura S, Miotto G (2007). Cytosolic LC3 ratio as a sensitive index of macroautophagy in isolated rat hepatocytes and H4-II-E cells.. Autophagy.

[28] Chan DC (2006). Mitochondria: dynamic organelles in disease, aging, and development.. Cell.

[29] Rossignol R, Gilkerson R, Aggeler R, Yamagata K, Remington SJ (2004). Energy substrate modulates mitochondrial structure and oxidative capacity in cancer cells.. Cancer Res.

[30] Marroquin LD, Hynes J, Dykens JA, Jamieson JD, Will Y (2007). Circumventing the Crabtree effect: replacing media glucose with galactose increases susceptibility of HepG2 cells to mitochondrial toxicants.. Toxicol Sci.

[31] Saraste M (1999). Oxidative phosphorylation at the fin de siecle.. Science.

[32] Diaz G, Liu S, Isola R, Diana A, Falchi AM (2003). Mitochondrial localization of reactive oxygen species by dihydrofluorescein probes.. Histochem Cell Biol.

[33] Distelmaier F, Koopman WJ, Testa ER, de Jong AS, Swarts HG (2008). Life cell quantification of mitochondrial membrane potential at the single organelle level.. Cytometry A.

[34] Liu X, Kim CN, Yang J, Jemmerson R, Wang X (1996). Induction of apoptotic program in cell-free extracts: requirement for dATP and cytochrome c.. Cell.

[35] Kim I, Rodriguez-Enriquez S, Lemasters JJ (2007). Selective degradation of mitochondria by mitophagy.. Arch Biochem Biophys.

[36] James DI, Parone PA, Mattenberger Y, Martinou JC (2003). hFis1, a novel component of the mammalian mitochondrial fission machinery.. J Biol Chem.

[37] Fransson S, Ruusala A, Aspenstrom P (2006). The atypical Rho GTPases Miro-1 and Miro-2 have essential roles in mitochondrial trafficking.. Biochem Biophys Res Commun.

[38] Frederick RL, McCaffery JM, Cunningham KW, Okamoto K, Shaw JM (2004). Yeast Miro GTPase, Gem1p, regulates mitochondrial morphology via a novel pathway.. J Cell Biol.

[39] Santel A, Frank S, Gaume B, Herrler M, Youle RJ (2003). Mitofusin-1 protein is a generally expressed mediator of mitochondrial fusion in mammalian cells.. J Cell Sci.

[40] Koshiba T, Detmer SA, Kaiser JT, Chen H, McCaffery JM (2004). Structural basis of mitochondrial tethering by mitofusin complexes.. Science.

[41] Tanaka Y, Kanai Y, Okada Y, Nonaka S, Takeda S (1998). Targeted disruption of mouse conventional kinesin heavy chain, kif5B, results in abnormal perinuclear clustering of mitochondria.. Cell.

[42] Cai Q, Sheng ZH (2009). Moving or stopping mitochondria: Miro as a traffic cop by sensing calcium.. Neuron.

[43] Chou T, Kim KS, Oster G (2001). Statistical thermodynamics of membrane bending-mediated protein-protein attractions.. Biophys J.

[44] Reynwar BJ, Illya G, Harmandaris VA, Muller MM, Kremer K (2007). Aggregation and vesiculation of membrane proteins by curvature-mediated interactions.. Nature.

[45] Hofmann MW, Weise K, Ollesch J, Agrawal P, Stalz H (2004). De novo design of conformationally flexible transmembrane peptides driving membrane fusion.. Proc Natl Acad Sci U S A.

[46] Ollesch J, Poschner BC, Nikolaus J, Hofmann MW, Herrmann A (2008). Secondary structure and distribution of fusogenic LV-peptides in lipid membranes.. Eur Biophys J.

[47] Lyamzaev KG, Izyumov DS, Avetisyan AV, Yang F, Pletjushkina OY (2004). Inhibition of mitochondrial bioenergetics: the effects on structure of mitochondria in the cell and on apoptosis.. Acta Biochim Pol.

[48] Mitra K, Wunder C, Roysam B, Lin G, Lippincott-Schwartz J (2009). A hyperfused mitochondrial state achieved at G1-S regulates cyclin E buildup and entry into S phase.. Proc Natl Acad Sci U S A.

[49] Lee YJ, Jeong SY, Karbowski M, Smith CL, Youle RJ (2004). Roles of the mammalian mitochondrial fission and fusion mediators Fis1, Drp1, and Opa1 in apoptosis.. Mol Biol Cell.

[50] Ferrara N (2004). Vascular endothelial growth factor as a target for anticancer therapy.. Oncologist.

[51] Filippova GN, Thienes CP, Penn BH, Cho DH, Hu YJ (2001). CTCF-binding sites flank CTG/CAG repeats and form a methylation-sensitive insulator at the DM1 locus.. Nat Genet.

[52] Cho DH, Thienes CP, Mahoney SE, Analau E, Filippova GN (2005). Antisense transcription and heterochromatin at the DM1 CTG repeats are constrained by CTCF.. Mol Cell.

[53] Stadhouders AM, Jap PH, Winkler HP, Eppenberger HM, Wallimann T (1994). Mitochondrial creatine kinase: a major constituent of pathological inclusions seen in mitochondrial myopathies.. Proc Natl Acad Sci U S A.

[54] He TC, Zhou S, da Costa LT, Yu J, Kinzler KW (1998). A simplified system for generating recombinant adenoviruses.. Proc Natl Acad Sci U S A.

[55] Chartier C, Degryse E, Gantzer M, Dieterle A, Pavirani A (1996). Efficient generation of recombinant adenovirus vectors by homologous recombination in Escherichia coli.. J Virol.

[56] Schiedner G, Hertel S, Kochanek S (2000). Efficient transformation of primary human amniocytes by E1 functions of Ad5: generation of new cell lines for adenoviral vector production.. Hum Gene Ther.

[57] Graham FL, Smiley J, Russell WC, Nairn R (1977). Characteristics of a human cell line transformed by DNA from human adenovirus type 5.. J Gen Virol.

[58] Koopman WJ, Distelmaier F, Esseling JJ, Smeitink JA, Willems PH (2008). Computer-assisted live cell analysis of mitochondrial membrane potential, morphology and calcium handling.. Methods.

[59] Yla-Anttila P, Vihinen H, Jokitalo E, Eskelinen EL (2009). Monitoring autophagy by electron microscopy in Mammalian cells.. Methods Enzymol.

